# Targeting Host Innate and Adaptive Immunity to Achieve the Functional Cure of Chronic Hepatitis B

**DOI:** 10.3390/vaccines8020216

**Published:** 2020-05-11

**Authors:** Sayeh Ezzikouri, Mohammad Enamul Hoque Kayesh, Soumaya Benjelloun, Michinori Kohara, Kyoko Tsukiyama-Kohara

**Affiliations:** 1 Virology Unit, Viral Hepatitis Laboratory, Institut Pasteur du Maroc, Casablanca 20250, Morocco; soumaya.benjelloun@pasteur.ma; 2 Transboundary Animal Diseases Centre, Joint Faculty of Veterinary Medicine, Kagoshima University, Kagoshima 890-0065, Japan; mehkayesh@pstu.ac.bd; 3 Department of Microbiology and Public Health, Faculty of Animal Science and Veterinary Medicine, Patuakhali Science and Technology University, Barishal 8210, Bangladesh; 4 Department of Microbiology and Cell Biology, The Tokyo Metropolitan Institute of Medical Science, Tokyo 156-8506, Japan; kohara-mc@igakuken.or.jp

**Keywords:** HBV, innate immunity, adaptive immunity, host factors, therapeutic vaccine

## Abstract

Despite the availability of an effective preventive vaccine for hepatitis B virus (HBV) for over 38 years, chronic HBV (CHB) infection remains a global health burden with around 257 million patients. The ideal treatment goal for CHB infection would be to achieve complete cure; however, current therapies such as peg-interferon and nucleos(t)ide analogs are unable to achieve the functional cure, the newly set target for HBV chronic infection. Considering the fact functional cure has been accepted as an endpoint in the treatment of chronic hepatitis B by scientific committee, the development of alternative therapeutic strategies is urgently needed to functionally cure CHB infection. A promising target for future therapeutic strategies is immune modulation to restore dysfunctional HBV-specific immunity. In this review, we provide an overview of the progress in alternative therapeutic strategies, including immune-based therapeutic approaches that enhance host innate and adaptive immunity to achieve and increase the functional cure from CHB infection.

## 1. Introduction

Hepatitis B virus (HBV) is an enveloped, circular, and partially double stranded relaxed circular DNA (rcDNA) virus that replicates by the reverse transcription of an RNA intermediate [1,2]. This rcDNA is converted into a covalently closed circular DNA (cccDNA) in the host cell nucleus. HBV is the prototype member of the Hepadnaviridae family and is one of the smallest enveloped DNA viruses [3], with a genome of approximately 3.2 kilobase pairs containing four overlapping open reading frames (ORFs), encoding seven proteins: polymerase/reverse transcriptase (RT), capsid-forming core protein, secreted precore protein (also known as hepatitis B e-antigen (HBeAg)), three related envelope proteins (HBV surface proteins (HBs) large, middle, and small), and a regulatory X protein [4,5,6]. The envelope and core proteins are structural proteins, whereas X protein and polymerase are nonstructural proteins [6,7,8]. Based on genome sequence divergence (>8%), HBV is classified into ten genotypes, A–H, that display different geographical distributions [9,10,11] and affect disease course and outcomes with current treatment strategies, alongside genetic variants [12].

HBV infection is a major global public health concern as it can cause a range of liver illnesses, from acute (including fulminant hepatic failure) to chronic hepatitis, cirrhosis, and hepatocellular carcinoma (HCC) [13]. Indeed, an estimated 257 million HBV infections occur globally each year [14], causing 887,000 deaths mostly due to cirrhosis and HCC [14]. Despite an effective vaccine having been available since 1980, HBV continues to be an endemic in many countries, and chronic HBV infection (CHB) is the leading cause of HCC, underlying around 50% of the HCC cases worldwide [15,16,17]. CHB carriers may pass through disease phases based on their serological profile and HBV DNA and alanine aminotransferase (ALT) levels [18]: (1) HBeAg-positive chronic infection; (2) HBeAg-positive chronic hepatitis; (3) HBeAg-negative chronic infection; (4) HBeAg-negative chronic hepatitis; and (5) the HBsAg-negative phase [18]. The first phase, previously known as the “immune tolerant” phase, is characterized by the presence of HBeAg, very high serum HBV DNA levels, and normal ALT levels. The phase is “HBeAg-positive chronic hepatitis B” phase is characterized by the presence of serum HBeAg, high HBV DNA levels, and elevated ALT levels. The third phase, previously termed the “inactive carrier” phase, is characterized by the presence of serum antibodies against HBeAg (anti-HBe), undetectable or low HBV DNA levels (<2000 IU/mL), and normal ALT. The fourth phase is characterized by a lack of serum HBeAg, usually with detectable anti-HBe, persistent or fluctuating moderate-to-high serum HBV DNA levels, and fluctuating or persistently elevated ALT levels. The final phase, also known as occult HBV infection, is observed in patients who clear HBsAg and antibodies against HBcAg (anti-HBc), with or without detectable antibodies against HBsAg (anti-HBs), and have undetectable serum HBV DNA levels; however, HBV DNA persists in the serum of some patients and in the liver of all patients [18]. Unfortunately, the mechanisms that drive the HBV progression via these distinct clinical phases to end-stage liver disease are poorly understood [19].

There are currently two classes of clinically available drugs to treat CHB infection. The first is based on the sub-cutaneous administration of pegylated interferon alpha (Peg-IFNα) which results in HBeAg seroconversion in 24–27% of patients and HBsAg loss in 3–7% of patients [20,21].

The second is of the six third-generation approved nucleos(t)ide analogs (NAs), including three nucleoside analogs (lamivudine, entecavir, and telbivudine) and three nucleotide analogs (adefovir, tenofovir disoproxil, and tenofovir alafenamide). NAs inhibit the RNA-dependent DNA polymerase reverse transcriptase with negligible adverse effects and can induce HBeAg and HBsAg loss in 12–22% and 0–3% of patients, respectively, after 48–52 weeks of treatment [20,21]. Since entecavir, tenofovir disoproxil, and tenofovir alafenamide are associated with a significantly lower risk of drug resistance than lamivudine and adefovir (older agents), they are considered first-line treatments [22]. NAs also partially restore virus-specific T cell responses and may increase the likelihood of complete HBV control [23], whereas Peg-IFNα is less effective at suppressing viral replication than the efficacy exhibited by NAs and is often associated with severe side effects [20]. Although NAs and Peg-IFNα can suppress HBV replication and reduce liver inflammation linked with cirrhosis, they cannot completely cure HBV infection, due to the persistence of cccDNA and/or integrated HBV DNA in hepatocytes. Thus, treatment responses are generally not durable and patients may experience HBV reactivation upon treatment withdrawal or immune-suppressive and biological therapies [22,24,25].

A recent consensus workshop conducted by the European Association for the Study of the Liver (EASL) and the American Association for the Study of Liver Diseases (AASLD) set the acceptable HBV treatment endpoints as functional cure. This includes HBsAg loss with or without hepatitis B surface antibody seroconversion and the persistence of transcriptionally inactive cccDNA 24 weeks after therapy, which are associated with improved clinical outcomes in a higher proportion of patients than those achieved with existing treatments [20,26]. Since a complete sterilizing cure involving viral eradication is unlikely to be feasible [20], it is crucial to investigate alternative therapeutic approaches to develop a functional cure for CHB infection [20,26].

The outcomes of HBV infection largely rely on virus–host interactions; for instance, 95% of immunocompetent adults can clear HBV infection, compared to only 5–10% of children [27]. Thus, timely and coordinated innate and adaptive immune responses are necessary to sense and control HBV infection [20]. CHB infection impairs virus-specific T cell responses by endowing specific T cells with exhausted phenotypes, poor effector cytotoxic activity, impaired cytokine production, and sustained expression of inhibitory receptors including programmed cell death-1 (PD-1), lymphocyte-activation gene-3 (LAG-3), cytotoxic T lymphocyte-associated antigen (CTLA)-4, and cluster of differentiation (CD) 244 [28]. The effector functions of the HBV-specific adaptive T cell response and neutralizing antibody development play key roles in the outcome of HBV infection and their dysregulation may lead to CHB [23].

An improved understanding of the mechanisms underlying immunological HBV tolerance is needed to develop effective immunotherapeutic strategies to cure CHB infection and prevent negative patient outcomes [23]. Since currently available treatment regimens are often unable to functionally cure CHB infection, combinations of antiviral(s) and immune modulatory therapies should be investigated [26], with the elimination of cccDNA and integrated viral DNA posing important challenges. This review summarizes current progress in immune-based therapeutic approaches and efficacy data obtained from different preclinical and clinical studies of CHB infection. In addition, we highlight the importance of boosting innate and adaptive immunity in combination with existing therapies to increase the functional cure rate of CHB.

## 2. Immune Restoration Induced by Peg-IFNs

IFN-α has been used to treat CHB since 1976 [29] and was first licensed to treat CHB in 1991. Peg-IFN-α2b and -α2a, which were introduced in 2005 to replace standard IFN due to their improved pharmacokinetic properties [30], have antiviral and immunomodulatory properties against HBV infection [31,32]; however, the exact mechanisms underlying their antiviral effects are not fully understood. Although randomized controlled trials have demonstrated the efficacy of Peg-IFNs for treating CHB [33,34,35,36,37], they only achieve viral clearance in 10–20% of Caucasians and <5% of Asian patients [38].

Peg-IFNα monotherapy is a treatment recommended by the Chinese international clinical practice treatment guidelines to help more CHB patients achieve a sustained off-treatment virological response and prevent unfavorable events such as cirrhosis and HCC [39]. Common causes of Peg-IFNα treatment discontinuation are adverse effects (AEs), including fatigue, emotional lability, alopecia, pyrexia, autoimmune disease, bone marrow suppression, insomnia, depression-related events, and poor tolerability in some individuals [40,41,42]. To avoid these systemic side effects of IFN-α that limit its clinical use, studies have investigated other means of inducing an intrahepatic antiviral immune response in the infected host [27].

Previous studies have reported that IFN-α induces specific IFN-stimulated genes (ISGs) and the secretion of effector proteins with antiviral properties against HBV infection [43,44,45]. The effects of IFN-α are partly mediated by the epigenetic repression of HBV cccDNA transcriptional activity [46] and it has been reported that IFN-α can inhibit HBV replication indirectly by driving proliferation, activation, and antiviral potential of natural killer (NK) cells in vivo [47,48,49]. Treatment with Peg-IFNα followed by sequential NAs has been associated with a greater decline in HBsAg and improved NK cells function [49]. Interestingly, Lucifora et al. demonstrated that IFN-α and the putative antiviral cytokine, lymphotoxin-β receptor (LTβR) could induce cccDNA degradation in an infectious cell culture system without detectable hepatotoxicity [50]. They also showed that IFN-α and LTβR agonists triggered APOBEC3A and APOBEC3B expression, respectively, resulting in the selective deamination of the cccDNA minus-strand and thus its degradation [50,51]. Xia et al. found that the T cell-derived cytokines IFN-γ and tumor necrosis factor (TNF)-α synergistically induced APOBEC3A and APOBEC3B [52,53]. Since these data indicate that IFN-α acts at multiple stages of the HBV life cycle, further studies should investigate the mechanisms underlying the anti-HBV effects of IFN-α to suggest new avenues for more effective HBV elimination [51].

## 3. Innate Immune Response Modulation

Since the majority of immunocompetent adults can clear HBV infection spontaneously, host immune responses may play important roles in controlling HBV infection. Indeed, coordinated interplay between innate and adaptive immunity has been shown to mediate the clearance of HBV infection [19,54,55]. CHB development is associated with strong innate immune response impairment, the age of infection, and the failure of HBV-specific immune responses; thus, the rescue of exhausted T cell reactivity may represent a rational strategy for curing HBV infection [56,57,58,59,60]. Patients who can control and clear HBV infection generally display robust helper and cytotoxic T cell responses against different viral antigens and their B cells produce anti-HBs antibodies [61,62,63].

The innate immune system is an important component of host immunity that consists of physical barriers, various phagocytic cells, cytokines, IFNs, and ISGs, which provide first line defense mechanisms and trigger specific and robust adaptive immunity against many viral infections [64,65]. However, most viruses have evolved efficient mechanisms to evade the innate system and establish infection [66,67]. Pattern recognition receptors (PRRs) are the major sensors of exogenous pathogens and include Toll-like receptors (TLRs), retinoic acid-inducible gene I (RIG-I), nucleotide oligomerization domain (NOD)-like receptors (NLRs), and melanoma differentiation-associated gene 5 (MAD5) [68]. In animal models, HBV infection does not induce an intrahepatic IFN/ISG response, suggesting that HBV acts as a stealth virus and induces a negligible innate immune response during the early phase of infection [69,70,71]; thus, HBV may not be detected by PRRs in infected cells. Recent data suggest that HBV is invisible to PRRs on hepatocytes [72], while in vitro data and chimeric mice showed that weak-to-moderate IFN and ISG up-regulation and did not actively interfere with the intrinsic innate immunity of infected hepatocytes [73,74,75,76,77]. Sato et al. showed that RIG induced an IFN response during HBV infection and directly exerted antiviral activities by blocking HBV polymerase from binding to viral pre-genomic RNA (pgRNA) to suppress viral replication [75]. Thus, molecules that target immune responses hold a great promise for curing CHB, particularly those that target PRR-mediated innate immunity in HBV-infected hepatocytes to trigger the antiviral mechanisms of liver nonparenchymal cells (Table 1).

### 3.1. TLR7 Agonists

One major area of focus for CHB therapies has been the development of synthetic agonists targeting PRRs, such as TLRs. TLR7 is expressed in a subset of human immune cells, primarily plasmacytoid DCs (pDCs) and B lymphocytes, and is located within the endosome where it recognizes nucleic-acid-like structures and small molecule agonists [97]. TLR7 activation results in B cell polyclonal expansion and differentiation toward immunoglobulin-producing plasma cells to support the humoral response [98,99]. TLR7 activation can also augment antigen processing and presentation and thus T cell responses, up-regulate costimulatory molecules critical for cytotoxic CD8+ T cell cross-priming [100], and can induce the production of various cytokines and chemokines [101,102,103]. In vitro models such as primary human hepatocytes (PHH) and differentiated HepaRG (dHepaRG) cells have revealed that GS-9620 can durably suppress HBV by inducing IFN without reducing cccDNA levels to enhance HBV antigen presentation [104]. These data provide proof-of-concept that TLR7 agonism can drive a sustained immune control to functionally cure CHB.

In preclinical studies in chimpanzees and woodchucks, the oral TLR7 agonist GS-9620 provided long-term suppression of serum and liver viral DNA and serum HBsAg or HBeAg levels [105,106], while also inducing the production of IFN-α and other cytokines and chemokines, up-regulating ISG expression, and activating the NK, CD8+ T, and B cells [105,106,107]. Preliminary clinical trial data have shown that GS-9620 can induce ISG15 without serum IFN and augment T and NK cell responses at low doses; however, no antiviral efficacy was observed as single agent or in combination with NAs, despite being safe and well-tolerated [77,78,79,80,106]. Since GS-9620 may have been capped at a suboptimal dose due to patient tolerability issues [80], a novel orally-administered TLR7 agonist (APR002) was designed and optimized [108]. Weekly oral doses of APR002 in woodchucks chronically infected with woodchuck hepatitis virus (WHV) were well-tolerated and yielded similar TLR7 activity with a distinct pharmacological profile [109].

### 3.2. TLR8 Agonist

TLR8 is mainly expressed in monocytes and myeloid DCs (mDCs), where induce their activation toward a strong Th1 profile; indeed, Jo et al. showed that stimulation with a TLR8 agonist induces a potent hepatic innate immune response [110]. GS-9688 (Selgantolimod) is an orally-active, potent, and selective small molecule TLR8 agonist that is well-tolerated and induces a sustained antiviral response in a woodchuck model of CHB [111]. In addition, GS-9688 reduced HBV DNA, RNA, and antigen levels in HBV-infected PHH [81] and induced TNF-α, IFN-γ, IL-12, IL-18, TNF-α, and IFN-λ expression in PBMC cultures with the potential to modify regulatory cell subsets as part of a Phase 2 multicenter, randomized, double-blind study [81]. GS-9688 was well-tolerated over an extended dosing period in combination with oral antivirals and demonstrated dose-dependent pharmacodynamic activity, with 5% of the GS-9688-treated patients achieving a ≥1 log10 IU/mL decline in HBsAg levels or HBeAg loss at 24 weeks [81].

### 3.3. RIG-I Agonist

The RIG-I agonist SB9200 (Inarigivir), a nucleotide-binding oligomerization domain-containing protein 2, is an oral HBV antiviral with both direct activity and immune-modulating activity via RIG-I [112,113]. In a chronic WHV model, SB9200 was shown to reduce serum WHV DNA and markedly reduce hepatic WHV and WHV surface antigens after at 12 weeks of treatment [109,114]. These antiviral effects were associated with the temporal induction of IFN-α, IFN-β, and ISGs in the blood and liver [109,114]. In the placebo controlled ACHIEVE trial of ascending SB9200 doses daily for 12 weeks, low doses were well-tolerated and associated with reduced HBV DNA and RNA levels [82,115]. These effects were observed without fully activating the immune response, consistent with a direct anti-viral effect that may involve targeting HBV RNA encapsidation [115]. Furthermore, the toxicity profile of Inarigivir was deemed to be mild and most AEs were mild to moderate [82,115].

### 3.4. Thymosin α1 (Tα1)

Tα1 (thymalfasin, Zadaxin) is a peptide consisting of 28 amino acids that was originally isolated from the thymus and promotes the reconstitution of immune defects by acting as an endogenous regulator of both the innate and adaptive immune systems [116]. Its mechanism of action has been hypothesized to be related to its immunomodulatory activities [116,117,118], while it has been shown that Tα1 acts via TLR2 and TLR9 in myeloid cells and pDCs, leading to DC and T cell activation and differentiation, as well as the expression of cytokines such as IFN-γ and interleukin-2 (IL-2) [119].

In a chronic WHV model, Tα1 treatment was associated with reduced viremia, while clinical trials have shown that Tα1 is effective in CHB patients with fewer side effects than IFN-α and may be preferable in Asian patients (reviewed in [83]). In combination with entecavir (ETV), Tα1 yielded similar virological, serological, and biochemical responses to ETV monotherapy at 104 weeks in patients with compensated liver cirrhosis, while both therapies were well-tolerated [120]; however, combination therapy had a higher tendency to inhibit HCC development than ETV treatment alone [120].

In summary, preclinical and clinical studies have shown that the intrahepatic activation of PRR-mediated innate immune responses by agonists efficiently controls HBV infection and restores antiviral immunity. In combination with FDA-approved HBV drugs, these direct-acting agents may help to functionally cure CHB infection; however, further clinical studies are required to prevent immune response overdosing in CHB patients and uncontrolled liver damage. Thus, comprehensive investigations will be needed to optimize dosages and identify patients that will achieve high antiviral efficacy with minimal AEs.

## 4. Adaptive Immune Response Modulation

In CHB patients, HBV-specific T cell responses are characterized by functional exhaustion, premature deletion, and weak virus-specific T cell responses that impede viral clearance, while the presence of HBV-specific-antibody producing B cells and functional HBV-specific T cells ultimately determines the outcome of HBV infection. These concepts have been analyzed in several previous reviews [56,121,122,123,124,125]. T cells exhausted by persistent exposure to high concentrations of antigen load, particularly HBsAg, are characterized by the hierarchical loss of effector T cell functions; upregulation of multiple inhibitory receptors known as checkpoint proteins, including PD-1, cell immunoglobulin mucin-3 (TIM-3), CD160, CTLA-4, LAG-3, and T-cell immunoreceptor with Ig and ITIM domain (TIGIT); impaired fatty acid oxidation (FAO); reactive oxygen species (ROS) overproduction; mitochondrial dysfunction; and defective effector T cells [28,125,126,127].

Although the existing preventive vaccine based on the HBs-S antigen is effective, it may be unable to prevent extracellular vesicle-mediated HBV infection [128]; therefore, it is important to target host immunity and reduce immune hyporesponsiveness/non-responsiveness or prevent HBV transmission to treat CHB infection. A promising novel approach for enhancing the efficacy of current antiviral therapy regimens is therapeutic vaccines, which have the potential to improve patient outcomes by inducing a dynamic immune response that can theoretically continue to adapt and expand following initial vaccination [129]. Although therapeutic vaccines have shown some success in animal models, these have not been observed in humans thus far [130]. Several types of therapeutic vaccine candidates are currently in development, including protein/peptide-based, live vector-based, DNA-based, and cell-based vaccine therapies [88] (Table 1).

### 4.1. PD-1/PD-L1 Blockade

The PD-1 receptor is mainly expressed on the surface of activated T and B cells, while its ligands PD-L1 and PD-L2 are found in a wide variety of cell types, including hepatocytes [131]. The PD-1/programmed death-1 ligand 1 (PD-L1) system may negatively regulate T cell function during HBV infection, with exhausted T cells being a hallmark of CHB [84]. The functional recovery of exhausted T cells is possible by blocking of immune checkpoints such as PD-1/PD-L1, which restores proliferation, cytokine secretion, and cytotoxic capability while decreasing viral load [132]. The in vitro blockade of woodchuck PD-L1 and PD-L2 during WHV antigen stimulation was found to improve virus-specific T cell responses in a subset of animals [133]. Moreover, ex vivo studies using blood from individuals with CHB infection have demonstrated that PD-1/PD-L1 blockade partially recovers dysfunctional virus-specific T and B cell responses [134,135,136,137]. In the chronic WHV model, anti-PD-L1 monoclonal antibody therapy (αPD-L1) combined with ETV treatment and therapeutic DNA WHV vaccination potently enhanced virus-specific T cell function, leading to the sustained immunological control of viral infection, anti-WHs antibody development, and complete viral clearance in some woodchucks [138]. Conversely, in a preclinical study of woodchucks, αPD-L1 monotherapy showed no efficacy; however, αPD-L1 plus ETV treatment reduced WHV DNA and WHsAg levels compared to ETV treatment alone, while both therapies were tolerable [139]. A recent study has shown that the inhibitory PD-1/PD-L1 axis is upregulated during CHB infection but not restored after successful antiretroviral therapy using NAs (undetectable viremia) [140]. In HBeAg-patients, treatment with monoclonal anti-PD-L1 antibodies (MEDI2790) increased HBV-specific T cell responses [140], while a phase Ib study found that the PD-1 inhibitor nivolumab (single dose, 0.1 or 0.3 mg/kg) was safe and well-tolerated with or without a therapeutic vaccine (GS-4774) [84] and decreased HBsAg titers by 0.5 log at 24 week, achieving sustained HBsAg loss and seroconversion in one patient [84].

### 4.2. Protein/Peptide-Based Vaccines

GS-4774 is a yeast-based therapeutic vaccine engineered to express HBV antigens (S, core, and X proteins). In a phase II study, GS-4774 showed no significant reduction in serum HBsAg [85]; however, GS-4774 increased HBV-specific T-cell responses in CHB when combined with tenofovir without significantly decreasing HBsAg levels [86].

A therapeutic vaccine candidate containing both HBsAg and core antigen (HBcAg), NASVAC reduced serum viral DNA levels more than Peg-IFN treatment in an open, phase III, randomized, treatment-controlled clinical trial [141]. Moreover, a recent study in a *Tupaia* model demonstrated that that intranasal immunization with HBs-S or HBs-L combined with HBc and formulated with carboxyl vinyl polymer (CVP) could induce strong IgG, IgA, neutralizing antibody, and HBV protein-specific IFN-γ immune responses [87].

DV-601, a therapeutic vaccine composed of HBs and HBc antigens, was found to be safe and well-tolerated with an adjuvant in a phase 1 study and produced antiviral response [89].

HepTcell, is an immunotherapeutic synthetic product composed of nine peptides (from highly conserved regions in three different HBV antigens (polymerase, core, and surface) designed to stimulate CD4+ and CD8+ T cells in HBV carriers irrespective of their HLA background. In a phase 1 study, HepTcell immunotherapy with an IC31 (TLR9 agonist) adjuvant was found to be well-tolerated and increased T cell responses against HBV with no observable effect on HBsAg [90].

### 4.3. Live Vector-Based Vaccines

TG1050 is an adenovirus -based therapeutic vaccine that expresses three HBV proteins (polymerase, core, and surface antigen) and has shown immunogenicity and antiviral effects in mice [142]. In a phase 1 clinical trial in CHB patients receiving NA therapy, TG1050 displayed a good safety profile and induced an HBV-specific cellular immune, supporting further clinical evaluation, particularly in combination therapies [91]. Moreover, a phase 1 trial found that AIC649, an inactivated parapoxvirus (iPPVO) preparation, was well-tolerated and increased IL-1β, IL-6, IL-8, and IFN-γ levels while reducing IL-10 plasma levels [92].

### 4.4. DNA-Based Vaccines

DNA vaccination is becoming an exciting novel immunization approach and fast growing field in vaccine technology since its first reports at the beginning of the 1990s that plasmid DNA induces an immune response to the plasmid-encoded antigen [143,144]. Plasmid DNA can provide tissue expression of antigens over much longer periods of time, compared to the short half-life of injected protein antigens, and thus potentially prime the immune system better [145].

pCMV-S2.S is a DNA vaccine that encodes HBV small (S) and middle (preS2 +S) envelope proteins and was well-tolerated and capable of activating or restoring T cell responses in some CHB carriers in a phase I clinical trial of ten chronic HBV carriers; however, this effect was transitory and weak [93]. The efficacy of pCMV-S2.S DNA in preventing viral recurrence was later investigated in a phase I/II clinical trial of 70 patients that had been treated effectively with NAs for a median of three years. Although pCMV-S2.S was safe, it was incapable of controlling the recurrence and recovery of the anti-HBV immune response [94].

INO-1800 is a candidate HBV therapeutic DNA vaccine formed of DNA plasmids encoding HBsAg and HBcAg (Inovio Pharmaceuticals, Pennsylvania, United States) that has been evaluated in a phase I clinical trial of 90 NAs-treated participants. The trial investigated the safety profile and immunogenicity of dose combinations of INO-1800 and INO-9112 (DNA plasmid encoding human interleukin 12); however, the reports remain to be published (ClinicalTrials.gov, Identifier: NCT02431312).

HB-100 is another candidate adenoviral-based DNA vaccine that encodes S1/S2/S envelope genes, polymerase sequences, and HBV core and X proteins, with human IL-12 as an adjuvant. In a phase I study of 12 patients with CHB, HB-100 was administered with an oral antiviral (Adefovir) over a 48-week period; however, no significant HBeAg seroconversion was observed [146].

HB-110 is a second-generation adenoviral-based DNA vaccine adjuvanted with IL-12. In a phase I trial, 27 patients with CHB randomly received either adefovir dipivoxil (ADV) alone or in combination with HB-110. No adverse effects were observed following co-treatment with HB-110 and ADV, although Korean patients showed weaker HBV-specific T-cell responses and HBeAg seroconversion than those in Caucasian patients [96].

### 4.5. Cell-Based Therapies

Studies have shown that adoptive T cell therapies could be utilized to treat viral infections [147]; therefore, T cell vaccination could be a promising approach for CHB infection. A recent study demonstrated that the adoptive transfer of grafted T cells provides a promising novel therapeutic approach wherein the retroviral delivery of T cell chimeric antigen receptors (CARs) can enable primary human T cells to detect HBsAg-positive hepatocytes, release IFN-γ and IL-2, and lyse HBV replicating cells [148]. Another study found that T cells with a CAR specific for HBV envelope proteins could localize to the liver in mice, reducing HBV replication and only causing transient liver damage [149]; however, the adoption of this approach to treat infectious diseases has been met with considerable resistance [150].

In summary, HBV-specific T cells are quantitatively and functionally defective in CHB patients; however, immunotherapy may be able to reverse defective immune responses and reprogram HBV-specific T cells to functionally cure CHB infection. Although adaptive immune response modulators have had a limited success in a number of clinical trials, mostly achieving only partial T cell recovery, the global restoration of efficient HBV-specific T cell responses capable of targeting infected liver cells may be dangerous and must be tightly monitored. An improved understanding of T cell exhaustion heterogeneity may help to identify patient populations with exhausted T cell subsets that would be more responsive to immunomodulatory strategies.

## 5. Conclusions

Patients with CHB infection display a weak host immune response, with persistent HBV cccDNA, integrated HBV DNA in hepatocytes, dysfunctional T cells, and inadequate B cell responses acting as major barriers to curing HBV. Many different strategies can be used to improve innate immunity and functionally restore exhausted CD8 T cells to cure CHB infection. Several strategies, including antivirals targeting various stages of the HBV life cycle (e.g., HBV entry, viral replication, HBV cccDNA production, and viral protein expression) and immunotherapeutic agents are currently being explored in preclinical and clinical trials, and may have the potential to functionally cure CHB. To achieve this goal, an improved understanding is required of the factors that will limit the development of efficient antiviral immunity in different CHB phases and will provide more appropriate and successful combinations of immune modulators with highly effective NAs for optimal tolerability. Heterogenous patient populations are another challenge that may be overcome using personalized therapy. New immunological strategies targeting innate or adaptive immunity in patients with CHB infection may be more suitable for treating young patients in the HBeAg-positive CHB infection phase than older subjects in the active CHB phase [95,151]. Since younger patients display less compromised HBV-specific T-cell function and a lower pro-inflammatory response than older patients, they may be more likely to recover HBV-specific T-cell immunity and not develop severe inflammatory liver reactions [151]. However, the treatment of patients in the HBeAg-positive CHB infection phase remains a matter of debate.

## Figures and Tables

**Table 1:** Summary of immunomodulatory agents in clinical trials targeting HBV.

Compound Name	Class	Mechanism of Action	Clinical Phase	Effects on HBsAg/HBeAg	Other Data	Status	References
**Innate Immunity Activators**							
TLR7 (GS-9620, Gilead Sciences)	Small molecule	TLR7	II	No HBsAg decline in patients	Lack of effect on cccDNA in vitro. Dose-dependent induction of ISG15, NKs, and HBV-specific T cell response	Ongoing	[77,78,79,80]
TLR8 (GS-9688, Gilead Sciences)	Small molecule	TLR8	II	Decline in HBsAg levels or HBeAg loss at 24 weeks	Dose-dependent induction of IL-12, IL-18, TNF-α, and IFN-λ	Ongoing	[81]
RIG-I agonist (SB-9200, Springbank)	Small molecule	RIG-I	II	-	Dose-dependent antiviral response against HBV DNA and HBV RNA	Ongoing	[82]
Thymosin α1 (ZADAXIN, SciClone Pharmaceuticals)	Peptide	-	IV	Eliminated both HBsAg and HBeAg in select patients	Strong immune response	Ongoing	[83]
**Adaptive Immunity Activators**							
PD-1 inhibitor (Nivolumab, Opdivo, Bristol-Myers Squibb)	Monoclonal antibody	PD-1:PD-L1 inhibitor	I	Decrease in HBsAg titers	Single dose of Nivolumab (with or without GS4774)	Ongoing	[84]
GS-4774 (Gilead Sciences)	Heat-inactivated, yeast protein-based therapeutic vaccine (expressing HBsAg, HBcAg, HBx)	Vaccine	II	No significant reduction in HBsAg	Safe and well-tolerated	Discontinued	[85,86,87]
DV-601 (Dynavax Technologies)	Protein-based vaccine (containing HBsAg and HBcAg)	Vaccine	I	-	Produced antiviral response	Terminated	[88,89]
HepTcell (Altimmune)	Composed of nine peptides from highly conserved regions of HBV polymerase, core, and surface antigens	Vaccine	I	No effect on HBsAg	Well-tolerated, increased T cell responses against HBV over baseline compared to placebo	Ongoing	[90]
TG1050 (Transgene)	Adenovirus 5-based therapeutic vaccine expressing three HBV proteins (polymerase, core, and surface antigen)	Vaccine	I	-	Good safety profile, induced HBV-specific cellular immune response	Ongoing	[91]
AIC649 (AiCuris)	Inactivated parapoxvirus (iPPVO) preparation	Vaccine	I	-	Well-tolerated, increased IL-1β, IL-6, IL-8, IFN-γ and reduced IL-10 plasma levels	Ongoing	[92]
pCMV-S2.S (Institut Pasteur, France)	DNA-based vaccine encoding HBV small (S) and middle (preS2 +S) envelope proteins	Vaccine	I/II	-	Well-tolerated, activated or restored T cell responses in some CHB carriers, weak and transitory: incapable of controlling anti-HBV immune response recurrence and recovery	Ongoing	[93,94]
INO-1800 (Inovio)	Adenoviral-based DNA vaccine, encodes S1/S2/S envelope gene, core, polymerase sequences, X proteins and human IL-12 as adjuvant	Vaccine	I	-	Activated and expanded CD8+ killer T cells	Terminated	[88,95]
HB-110 (Genexine, Inc.)	Second-generation therapeutic HBV adenoviral-based DNA vaccine encoding S, L, core, polymerase protein, adjuvanted with IL-1	Vaccine	I	HBeAg seroconversion	Well-tolerated, induced weaker HBV-specific T cell responses in Korean patients than in Caucasian patients	Ongoing	[96]
